# Beyond Orbital Volume: Outcomes of Patient-Specific Implants, Surgical Navigation, and Artificial Intelligence in Orbital Reconstruction

**DOI:** 10.7759/cureus.117973

**Published:** 2026-10-15

**Authors:** Daniel Gravino, Michael Farrugia

**Affiliations:** 1 Surgery, Materdei, Msida, MLT; 2 Opthalmology, Mater Dei Hospital, Msida, MLT

**Keywords:** artificial intelligence, diplopia, enophthalmos, orbital fractures, orbital reconstruction, patient-reported outcomes, patient-specific implants, surgical navigation

## Abstract

Orbital reconstruction increasingly incorporates patient-specific implants (PSIs), surgical navigation, and artificial intelligence (AI), but the clinical value of these technologies depends on more than geometric accuracy. This critical narrative review examines their effects on globe position, binocular function, visual safety, and patient experience, principally in adult post-traumatic reconstruction.

Literature identification used targeted PubMed and Europe PMC searches, publisher sources, and citation tracing, covering publications through September 2026; targeted source verification during revision was undertaken on 10 October 2026. Selection was purposive rather than systematic. Earlier meta-analyses found inconsistent comparative benefits, whereas a newer systematic review reported favorable descriptive summaries for persistent enophthalmos, diplopia, and revision. These summaries should not be interpreted as pooled causal effect estimates. A small randomized trial supports improved volumetric accuracy with directly manufactured implants but does not establish durable functional superiority. Navigation may assist complex reconstruction, although co-interventions and selection bias limit attribution of benefit. A small contemporary polyether ether ketone (PEEK) implant cohort found no statistically demonstrable incremental navigation advantage.

AI primarily demonstrates performance in detection, segmentation, and virtual reconstruction, with postoperative benefit remaining uncertain. Outcome heterogeneity, incomplete reporting, and limited follow-up restrict comparisons. Selective use according to the reconstructive difficulty is reasonable, but proposed selection frameworks remain unvalidated. Prospective comparisons should measure standardized functional, patient-reported, safety, and economic outcomes alongside anatomical accuracy.

## Introduction and background

Orbital fractures can displace or entrap soft tissues, enlarge the bony cavity, and disturb the relationship between the globe and surrounding structures. Clinically important consequences include enophthalmos, meaning posterior globe displacement; hypoglobus, meaning inferior globe displacement; ocular motility restriction; and diplopia. Associated globe, retinal, or optic nerve injury may independently impair visual acuity. Conventional reconstruction releases displaced tissues and supports the defect using manually shaped or stock preformed implants. Residual deformity or implant malposition can compromise appearance, binocular function, and safety, sometimes requiring revision [1].

Patient-specific implants (PSIs) use the individual's imaging to customize implant geometry, either by shaping a stock implant on a patient-specific model or by directly manufacturing a bespoke implant. Navigation displays the position of tracked instruments relative to registered images and a planned reconstruction. Artificial intelligence (AI) can assist image interpretation, segmentation, and virtual reconstruction; it does not necessarily control implant placement. These technologies address different steps in the same treatment pathway and may be used independently [2,3].

Their evidence has evolved unevenly. Earlier pooled comparisons did not consistently demonstrate superiority, and the American Academy of Ophthalmology (AAO) classified all 19 studies in its PSI assessment as level III evidence [2,3]. More recent reviews describe promising clinical and technical results [4,5]. This review adds an outcome-centered appraisal across the complete pathway: what is being customized or guided, which endpoint improves, and whether the improvement can reasonably be attributed to the technology. Adult traumatic defects form the main evidence base; secondary reconstruction and other indications are considered separately.

## Review

Literature identification and selection

This is a critical narrative review, not a systematic review or a Preferred Reporting Items for Systematic Reviews and Meta-analyses (PRISMA)-based synthesis. The original literature identification used targeted searches of PubMed and Europe PubMed Central without a lower publication-date limit, supplemented by reference lists, PubMed Central full texts, and publisher websites. The publication window extended through September 2026. During revision, targeted web-assisted checks of indexed records, original articles, publisher sources, and an author-deposited full text were performed on October 10, 2026, including the two 2026 publications highlighted by the reviewers. This verification was not a new comprehensive database search.

The searches combined terms for the orbit (orbital or orbit) and reconstruction (reconstruction or fracture) with one or more technology terms: patient-specific, navigation, AI, or deep learning. Supplementary terms addressed diplopia, visual loss, quality of life, secondary reconstruction, and costs. Complete historical query logs, exact original execution dates, screening counts, and an exclusion log were not retained in the available review record. Appendix 1, therefore, provides reconstructed database-specific syntax for repeat searching, explicitly distinguished from a prospectively logged search history.

English-language clinical studies addressing reconstruction accuracy, globe position, ocular function, complications, and patient-reported outcomes in adults with traumatic defects were prioritized. Comparative studies and systematic reviews were used to assess effectiveness; uncontrolled series informed feasibility, unusual indications, and material-specific considerations. Computational studies were included for technical validation, and professional assessments for evidence appraisal. Studies confined to pediatric, congenital, or oncological reconstruction were not used to establish effectiveness in adult trauma. Reports without relevant clinical or technical outcomes were not central to the synthesis. Selection was purposive according to relevance, comparator quality, design, and outcome coverage, rather than based on a minimum effect size or statistical significance.

Bibliographic identification was not restricted to open-access publications. Access restrictions applied to full texts: Where unavailable, interpretation was limited to the indexed abstract and explicitly identified as such. Evidence was grouped by technology, study design, comparator, and outcome. Appraisal considered allocation, baseline comparability, masking, co-interventions, follow-up, measurement methods, and applicability, but no formal study-level risk-of-bias tool or certainty grading was applied. There was no registered protocol, independent duplicate screening, or new meta-analysis. Heterogeneous interventions and endpoints, inconsistent denominators, and overlapping cohorts made a new pooled estimate inappropriate. Published effect measures and uncertainty are reproduced where verified; missing information is not reconstructed from percentages.

Defining the intervention and reconstructive target

Terminology is standardized here as follows: a stock preformed implant has generic manufactured geometry; a freehand-contoured implant is shaped manually during surgery; a model-precontoured implant is shaped before surgery on a patient-specific model; and a directly manufactured PSI is fabricated or milled from a patient-specific digital design. When a source combines the latter two as PSIs, this grouping is stated. Comparator descriptions are retained rather than treating all non-PSI operations as equivalent [2,5].

Sigron et al. compared 17 model-precontoured reconstructions with 13 preceding freehand-contoured cases and reported operative times of 58.9 versus 94.8 minutes [6]. The sequential retrospective design cannot separate customization from experience or temporal change. These are operating-room durations, not complete planning-to-treatment times; engineering, model production, manufacturing, and waiting time require separate assessment. A review of model-based precontouring also identified substantial risk of bias in the underlying studies [7].

Virtual planning defines a target; navigation relates tracked instruments to that target; intraoperative imaging assesses the achieved reconstruction. Virtual planning is not inherently AI, and navigation is not interchangeable with imaging. Orbital volume is informative but incomplete: equal volumes can coexist with different local contours, implant positions, and soft-tissue relationships. The proposed importance of posterior support and the inferomedial transition is an anatomical rationale, not a validated independent predictor of functional recovery. Regional defect coverage and implant position should therefore complement global volumetry in research assessments [5].

Comparative evidence for PSIs

Kotecha et al. included 11 comparative studies and 628 adults, combining directly manufactured and model-precontoured PSIs [2]. Seven studies reported diplopia frequencies: 22/196 PSI patients and 47/245 conventional-treatment patients. These crude totals differ from the source's random-effects estimate: the relative risk for conventional versus PSI reconstruction was 1.90 (95% confidence interval (CI) 0.91-3.92; p = 0.07; I² = 0%). The interval allows a clinically important advantage but does not exclude no difference. Follow-up and diplopia definitions were not uniform, so these figures are not a common-time-point treatment effect.

Saptarshi et al. reviewed seven studies involving 560 participants and reported an enophthalmos advantage, but no statistically significant diplopia difference between PSI and standard mesh groups [8]. A nonsignificant postoperative volume difference between PSI and model-precontoured implants suggests that comparator quality matters; it does not establish equivalence.

Joachim et al. included 23 studies and 1,222 participants but explicitly did not perform a meta-analysis [4]. Their reported summary percentages favored PSIs for persistent enophthalmos (7.3% versus 18.2%; p = 0.03), diplopia (11.7% versus 30.1%; p = 0.01), and revision (5.9% versus 13.7%; p = 0.01). These are descriptive review-level summaries across heterogeneous studies, not verified patient-weighted crude risks or pooled adjusted effects. The diplopia comparison describes mean study rates in seven directly comparative studies. A common endpoint-specific patient denominator, standardized follow-up time, and comparative 95% CI are not provided alongside these summary percentages. They are retained as the source's reported findings, without deriving a risk ratio, absolute risk reduction, or number needed to treat. Successive reviews may reuse primary cohorts and are not independent clinical replications.

Sheta and Elsaadany randomized 28 patients with unilateral fractures, 14 per group, to directly manufactured titanium PSIs or model-precontoured titanium mesh [9]. Mean reconstructed-to-intact volume discrepancy was 0.6543 ± 0.2767 versus 1.666 ± 0.3884 cm³, respectively (reported p < 0.05; no comparative CI accompanying these abstract values). The power calculation concerned volumetry. Allocation used sealed envelopes; only the data analyst was blinded, and registration was retrospective (NCT06294535). Clinical follow-up extended to three months. Visual acuity was assessed using a standard distance chart, enophthalmos was measured with a Hertel exophthalmometer, and diplopia was assessed clinically by gaze examination, no detailed patient-reported functional scale was used. The trial supports anatomical accuracy but is too small and short to establish durable binocular benefit, cost-effectiveness, or rare-event safety.

Material and geometry must also be separated. Yang et al. reported improved orbital reconstruction in an uncontrolled 40-patient series using printed polycaprolactone-based PSIs [10]. This supports feasibility rather than superiority over other materials. The 2026 review by Yang and Sa adds contemporary fabrication and workflow context but is not an independent comparative cohort [5]. Material-specific durability requires surveillance beyond the early period, particularly when structural support changes during resorption.

Navigation and verification

Bly et al. compared 56 mirror-image-overlay-guided reconstructions with 57 cases without this guidance in a series of severe orbital fractures [11]. The favorable diplopia findings concern a computer-assisted pathway incorporating planning, endoscopy, and navigation, rather than an isolated navigation effect. Historical and treatment-selection differences remain plausible explanations for part of the observed benefit.

Verbist et al. included 27 studies and 696 patients, undertaking meta-analysis of volumetric accuracy and revision [12]. Their abstract reports revision frequencies of 4.9% with navigation and 17% with conventional surgery, but does not give endpoint-specific denominators, a common follow-up interval, or the comparative CI and model alongside those rates. The percentages are therefore treated as source-reported summaries, not a stand-alone pooled relative effect. Heterogeneity prevented pooling of several other endpoints. The AAO image-guidance assessment included 32 studies, of which two were level II and 30 level III [13]. Together, these sources suggest technical benefit in selected settings without securely isolating the contribution of navigation.

The 2026 retrospective service evaluation by de Oliveira Gorla et al. is particularly relevant because all ten patients received polyether ether ketone (PEEK) PSIs, with navigation in five and freehand placement in five [14]. The indexed abstract reports a between-group median difference in planned-treated volume discrepancy of 1.00 cm³ (95% CI -0.30 to 1.60; p = 0.151). Six of nine clinically evaluable patients met the study's success criteria, while three had persistent diplopia attributed to traumatic muscle injury. This small exploratory comparison did not demonstrate an incremental navigation advantage; it was not an equivalence study. It also does not establish PEEK superiority over another material.

Navigation can localize an instrument accurately relative to an inappropriate target. Registration error, segmentation error, displaced reference anatomy, and imperfect mirroring require separate checks. The display cannot confirm complete release of entrapped tissue. Navigation is therefore best understood as an adjunct to anatomical assessment, tissue protection, and verification of implant seating, with additional imaging used to answer a specific unresolved question [1,13].

AI applications and validation

Fracture Detection and Segmentation

Bao et al. reported an area under the curve of 0.992 for fracture detection and a Dice coefficient of 0.885 for segmentation under cross-validation [15]. Detection and segmentation are different tasks: discrimination concerns recognition, whereas Dice quantifies overlap with a labeled reference. Neither measures postoperative recovery.

Rashad et al. used 1,004 expert-segmented orbits in a multicenter study, reporting mean Dice coefficients of 0.935 for the orbit and 0.917 for the floor, with mean surface distances of 0.292 and 0.287 mm [16]. Eftimie et al. described automated segmentation and mirroring in 502 cranial CT scans, reporting a mean Dice coefficient of 0.936 and a mean symmetry-plane angular deviation of 0.95° in fractured cases [17]. Multicenter data collection alone does not establish independent external validation. The related investigators and similar dataset scale make cohort overlap possible; independence between these reports cannot be assumed without patient-level provenance. Published computational performance should not be counted as independent clinical replication.

Virtual Reconstruction

Yu et al. evaluated deep-learning virtual reconstruction using synthetic defects and a small clinical sample, including defects for which contralateral mirroring is problematic [18]. Validation addressed geometry and expert judgment rather than comparative postoperative outcomes. A plausible predicted surface does not establish the patient's original anatomy. Evaluation should separate synthetic testing from real traumatic defects and explicitly report institution-level external validation.

Clinical Outcome Prediction

Hassan et al. developed a diplopia risk calculator in 254 adults using logistic regression and a 70:30 development-validation split [19]. This is conventional statistical prediction with internal split-sample validation, not a deep-learning reconstruction system. Discrimination alone does not demonstrate calibration in another center or improvement in decisions and outcomes.

Requirements for clinical translation

Across applications, validation should document patient-level separation between training and testing, prevent leakage between scans from the same patient, and test performance in external institutions and scanners. High average overlap can conceal a clinically important local defect near the apex or posterior support. Regional surface error, unacceptable failure frequency, expert correction time, and performance in comminution, bilateral injury, and artifact-degraded scans should accompany global metrics. These are proposed requirements, not achievements established for every cited system. Clinical-validation guidance and image-analysis recommendations support distinguishing measurement agreement from decision utility and patient benefit [20-22].

Clinical outcomes and patient experience

Visual Acuity and Safety

Visual acuity, binocular function, and globe position are separate outcomes. Technology comparisons do not establish reversal of vision lost through retinal or optic nerve damage [3,13]. Future comparisons should record monocular best-corrected distance acuity using a stated chart and preferably logMAR reporting, document traumatic impairment before reconstruction, and assess early postoperative change. New loss should be distinguished from pre-existing injury through baseline examination, timing, identified mechanism, and adjudication; when baseline testing is impossible, this uncertainty must remain visible.

Jacobs et al. identified severe visual loss in 14 of 1,665 orbital operations across mixed indications [23]. This is contextual safety evidence, not a contemporary risk estimate for isolated fracture repair or PSI surgery. Complications may involve hemorrhage, implant malposition, ischemia, or direct injury. Small series without visual loss cannot establish safety for rare catastrophic events, and re-intervention decisions require clinical assessment beyond imaging symmetry [24].

Diplopia and Globe Position

Diplopia should be recorded in primary gaze, reading gaze, and other positions, with its effect on daily activities. New postoperative diplopia requires a denominator of patients without it at baseline; persistence and resolution require the baseline-symptomatic denominator. Hughes et al. reported residual diplopia in 4.9% of 472 reconstructions, while affected patients could still improve their binocular single-vision scores [25]. Binary persistence therefore need not imply absence of benefit.

Enophthalmos should be measured in millimeters using a specified exophthalmometer, consistent base settings and landmarks, with the contralateral side as a reference only when suitable. Displaced orbital rims can invalidate that reference. Hypoglobus requires a reproducible vertical reference plane and standardized head position; calibrated photographs or appropriately registered CT can complement examination. Continuous change and residual asymmetry should accompany prespecified thresholds, with patient-perceived appearance assessed separately. Table 1 proposes standardized follow-up rather than implying that these methods were applied uniformly in existing studies.

**Table 1:** Proposed outcome domains for comparative orbital reconstruction studies Author-developed proposal, not a validated consensus core outcome set. Time points are proposed for future research and do not imply routine imaging or uniform assessment in the cited studies.

Outcome Domain	Recommended measures	Suggested Timing and Reporting
Visual safety	Baseline; 3, 6, and 12 months. Prespecify meaningful change; report responders, missing data, and instrument limitations.	Baseline; early postoperative assessment; 3, 6, and 12 months. Separate traumatic impairment from new surgical loss and retain unassessable cases.
Binocular function	Primary-, reading-, and other-gaze diplopia; orthoptic examination and binocular single-vision field; impact on daily tasks.	Baseline; 3, 6, and 12 months. Separate new, persistent, resolved, and improved symptoms using appropriate baseline denominators.
Globe position	Enophthalmos and hypoglobus in mm; consistent exophthalmometer settings, reference landmarks, head position, and calibrated photographs.	Baseline after acute swelling is considered; 3, 6, and 12 months. Report continuous change and prespecified residual-deformity thresholds.
Anatomical accuracy	CT-based orbital volume discrepancy, regional surface error, implant position, and defect coverage; specify segmentation and registration methods.	Clinically or research-justified postoperative imaging. Document reference suitability and assessor masking; no automatic serial CT schedule.
Patient experience	Daily visual tasks, appearance, sensory symptoms, and generic or appropriately validated condition-specific measures.	Baseline; 3, 6, and 12 months. Prespecify meaningful change; report responders, missing data, and instrument limitations.
Durability and burden	Revision indication and timing, infection, exposure, migration, rehabilitation, and work absence.	Continuous event capture through 12 months and longer as indicated. Separate planned stages from unplanned procedures.
Resources and value	Total episode costs and resource quantities linked to functional or utility outcomes; specify perspective and comparator.	Track the full pathway and state time horizon. Include setup, training, manufacture, complications, and downstream care.

Quality of Life and Secondary Reconstruction

Rajantie et al. followed 26 surgically treated patients using the generic 15D instrument [26]. Mean scores were 0.898 preoperatively and 0.920 at six months, compared with 0.936 in the population comparator. The latter was a general-population reference, not a randomized alternative-treatment group. The six-month difference was nonsignificant, which does not establish equivalence or complete recovery. Interpretation requires a prespecified clinically important change and individual responder analysis as well as statistical testing; 19% still reported diplopia in daily life. Generic summary scores may obscure continuing appearance, sensory, or binocular problems.

Singh et al. reported improvement after computer-assisted secondary reconstruction in 33 previously operated patients [27]. Selection for revision, absence of a comparable untreated group, and concurrent tissue release limit attribution to the implant. The intended target should be stated: skeletal malposition, implant-related restriction, displaced soft tissue, or a combination. Symptom improvement in selected revision patients supports feasibility, not universal superiority of another reconstruction. A technically satisfactory skeleton with persistent muscle or nerve injury poses a different problem from a malpositioned implant.

Discussion

Comparative Effectiveness and Uncertainty

The most consistent signal concerns reconstruction geometry. Evidence for diplopia, globe position, and revision is promising but sensitive to study design, comparator quality, and outcome definition [2-4,9,12]. Evidence is insufficient to establish improved acuity, patient-reported recovery, or protection against rare harm; this is an absence of convincing evidence, not proof that the technologies cannot confer such benefits. General reviews of three-dimensional planning describe workflow applications but cannot independently support broad clinical superiority claims [28].

Confounding can operate in both directions. More difficult injuries may be allocated to PSIs, while patients able to wait for manufacture differ from urgent cases. New technologies may also be concentrated in experienced teams with better imaging and orthoptic support. Small heterogeneous pilot series illustrate applicability rather than comparative effectiveness [29]. Historical comparisons incorporate learning and changes in perioperative care. Future adjustment should include injury severity, baseline ocular function, injury-to-treatment interval, prior surgery, and surgeon or center effects.

Outcome denominators matter as much as implant categories. A group with fewer symptomatic patients before surgery may appear superior if all postoperative diplopia is combined. Revision should distinguish planned staging, early image-triggered correction, and later symptomatic failure. Attrition and follow-up duration affect opportunities to detect both recovery and complications. A positive multicomponent pathway study does not identify the value of every component.

Selecting Technology for the Reconstructive Problem

The following approach is author-developed and has not been prospectively validated as a clinical decision rule. Customized geometry is most plausibly useful when a stock implant cannot reproduce a large or multiwall defect reliably. Navigation addresses a different uncertainty: whether instruments and the implant are positioned according to the plan when landmarks are disrupted. Either may be used without the other. A limited defect with preserved support may be addressed using stock or model-precontoured reconstruction, depending on surgeon assessment and resources [8,11,14].

Manufacture must fit the clinically appropriate treatment window. The AAO PSI assessment described production times ranging from 1 to 35 days, mostly under 10 days [3]. These are context-dependent, not guaranteed turnaround times. Customization should not delay urgent management of threatened vision or clinically significant entrapment [1]. Adult unilateral trauma evidence does not establish equivalent benefit in bilateral injuries without an intact mirror reference, pediatric growth, congenital asymmetry, or oncological and irradiated composite defects. Oncological clearance and soft-tissue requirements must determine reconstruction rather than be constrained by a prefabricated target.

Outcomes and Economic Value

Table 1 is an author-developed reporting proposal, not a validated consensus core outcome set. Baseline assessment, early safety review, and functional follow-up at 3, 6, and 12 months would improve comparability; longer surveillance should reflect material and clinical context. Imaging should answer a clinical or research question rather than be repeated automatically at every visit.

Comparative studies should separate primary from secondary reconstruction and stratify defect complexity. Where equipoise exists, randomization can compare directly manufactured PSIs with contemporary model-precontoured implants, with navigation evaluated separately or in a feasible factorial design. Independent imaging review and blinded orthoptic assessment would reduce detection bias. Power calculations should concern a prespecified patient-important endpoint, not only a volume difference.

Economic analysis should compare resource use and patient outcomes over the whole episode of care. Relevant costs include engineering, manufacture, software, equipment, training, operating-room time, imaging, complications, reoperation, rehabilitation, and work absence. Shorter operating time alone does not establish cost-effectiveness. Incremental cost per meaningful functional improvement, or per quality-adjusted life-year when appropriate utility data are available, should be estimated against a stated comparator, perspective, and time horizon, with sensitivity analysis. Fewer revisions offset expenditure only if the difference is credible and relevant to the local service.

Limitations

Review-process limitations include restricted database coverage, purposive selection, English-language prioritization, incomplete full-text access, and unavailable historical screening logs. Embase, Scopus, Web of Science, and the Cochrane Library were not directly searched for this review. Relevant studies, including neutral or unfavorable findings, may have been missed. The reconstructed syntax improves transparency but cannot recreate an undocumented historical search. There was no independent duplicate screening, formal risk-of-bias assessment, or certainty grading; source-level appraisal is therefore interpretive and vulnerable to selection and interpretation bias.

Underlying-evidence limitations are distinct: small samples, nonrandom allocation, inconsistent masking, co-interventions, varied comparators, heterogeneous definitions and follow-up, and limited reporting of patient-reported outcomes and rare harm. Successive reviews may contain overlapping cohorts; related AI reports may also share data. Abstract-only interpretation restricts verification of denominators and statistical methods for some comparisons, including the PEEK service evaluation and the navigation review. These limitations prevent firm conclusions about universal superiority, durable functional benefit, or economic value outside the populations studied.

## Conclusions

Comparative evidence supports improved anatomical accuracy with some patient-specific and navigation-assisted approaches, but the magnitude and clinical consequences remain dependent on the intervention, comparator, and setting. Reported reductions in diplopia, globe malposition, and revision are encouraging rather than definitive evidence of universal functional superiority. Visual acuity, binocular function, appearance, and patient experience must be assessed separately. AI has demonstrated computational capability, while postoperative benefit remains unproven. Prospective comparisons with contemporary controls and standardized functional, patient-reported, safety, and economic outcomes are needed to determine which patients benefit and whether additional resources are justified.
